# Impact of seasonal malaria chemoprevention on hospital admissions and mortality in children under 5 years of age in Ouelessebougou, Mali

**DOI:** 10.1186/s12936-020-03175-y

**Published:** 2020-03-03

**Authors:** Djibrilla Issiaka, Amadou Barry, Tiangoua Traore, Boubacar Diarra, David Cook, Mohamed Keita, Issaka Sagara, Patrick Duffy, Michal Fried, Alassane Dicko

**Affiliations:** 1 Malaria Research & Training Center, Faculty of Pharmacy and Faculty of Medicine and Dentistry, University of Science Techniques and Technologies of Bamako, Bamako, Mali; 2 Centre de Santé de Reference de Ouelessebougou, Ouelessebougou, Mali; 3 Laboratory of Malaria Immunology and Vaccinology, National Institute of Allergy and Infectious Diseases, Bethesda, MD USA; 4 Programme National de Lutte contre le Paludisme, Bamako, Mali

**Keywords:** Malaria, Seasonal malaria chemoprevention, Hospitalization, Death, Mali

## Abstract

**Background:**

Seasonal malaria chemoprevention is widely implemented in Sahel and sub-Sahel countries in Africa. Few studies have assessed the impact of the SMC on hospital admission and death when it is implemented in the health system. This retrospective study assessed the impact of seasonal malaria chemoprevention (SMC) on hospitalizations and deaths of children under 5 years of age during the second year of implementation of SMC in the health district of Ouelessebougou in Mali.

**Methods:**

In February 2017, a survey was conducted to assess hospital admissions and deaths in children under 5 years of age in two health sub-districts where SMC was implemented in 2015 and two health sub-districts where SMC was not implemented. The survey reviewed deaths and hospitalizations of children under 5, in the four health sub-districts. The crude and specific incidence rates of hospitalizations and deaths were determined in both groups and expressed per 1000 children per year. A negative binomial regression model and a Cox model were used to estimate the relative risks of hospitalization and death after adjusting for confounders. The R software was used for data analysis.

**Results:**

A total of 6638 children under 5 years of age were surveyed, 2759 children in the SMC intervention areas and 3879 children in the control areas. All causes mortality rate per 1000 person-years was 8.29 in the control areas compared to 3.63 in the intervention areas; age and gender adjusted mortality rate ratio 0.44 (95% CI 0.22–0.91), p = 0.027. The incidence rate of all causes hospital admissions was 19.60 per 1000 person-years in the intervention group compared to 33.45 per 1000 person-years in the control group, giving an incidence rate ratio (IRR) adjusted for age and gender of 0.61 (95% CI 0.44–0.84), p = 0.003.

**Conclusion:**

The implementation of SMC was associated with a substantial reduction in hospital admissions and all-cause mortality.

* Trial registration* ClinicalTrials.gov NCT02646410.

## Background

In sub-Saharan Africa, over the last 15 years, the under-five mortality rate has decreased overall by 57% (95% CI 46% to 65%) [1]. Seasonal malaria chemoprevention (SMC), formerly known as intermittent preventive treatment in children (IPTc), is defined as intermittent administration of full treatment courses of an anti-malarial medicine, namely sulfadoxine–pyrimethamine and amodiaquine (SP–AQ) at monthly interval in children aged 3–59 months during the malaria season to prevent malarial illness [2]. SMC has been recommended in areas of high seasonal malaria transmission, particularly the Sahel region [2]. A meta-analysis showed a SMC protective efficacy of 57% against all-cause mortality (95% CI 24% to 76%), while a Cochrane review found a non-significant reduction of 44% (RR risk ratio of 0.66, 95% CI 0.31 to 1.39) in children aged 3 to 59 months [3].

Approximately 39 million children under 5 years of age live in areas of sub-Saharan Africa where SMC is deemed appropriate. In these areas, 33.7 million episodes of malaria and 152,000 deaths of children due to malaria are reported each year [4]. The results of estimates using statistical models suggest that SMC has the potential to avoid 21,217,125 malaria cases and 95,477 child deaths each year worldwide if successfully delivered to at-risk populations [4]. In Mali, the mortality rate among children under 5 was estimated at 95 per 1000 in 2014, with a higher risk of death for young children in rural areas than in urban areas (11.3% *versus* 6.4%) [5].

Mali phased in the implementation of SMC in 2012 in its health system, starting with the health district of Koutiala. In Ouelessebougou, the implementation of SMC began in 2014 in four sub-districts with the support of the National Malaria Control Programme in Mali (NMCP) and the Malaria Research and Training Center (MRTC) as part of the USAID-funded PEER-Health project through the United States National Academy of Science (https://sites.nationalacademies.org/PGA/PEER/PEERhealth/PGA_087173). In 2015, two sub-districts were added and by 2016 the entire district was covered. Our study evaluated the impact of SMC on hospitalization and mortality for children under 5 years of age in Ouelessebougou, 3 years after the start of the phased implementation in this health district.

## Methods

### Study site and procedure

The study was performed in the health district of Ouelessebougou. The health district of Ouelessebougou covers a total of 16 health sub-districts with a total population estimated at 260,351 inhabitants in 2016. About 84% of children under 5 years of age slept under impregnated bed nets the night before, based on a caregivers’ interview carried out in December 2016. Ouelessebougou is located in the circle of Kati, Koulikoro region in Mali. It is a sub-prefecture capital located 80 km south of Bamako. Ouelessebougou is in the seasonal malaria transmission zone of four to 5 months. Malaria is the primary cause of consultation (22%) in the study area [5]. The baseline malaria incidence rate in 2011 was two episodes per child per year; the in children under 5 years of age during the transmission season [6].

SMC began to be implemented in randomly-selected health areas of the Ouelessebougou health district in 2014. In both 2015 and 2016, two additional heath sub-districts each year were identified for SMC administration. This provided the opportunity to compare the two areas that received SMC in 2015 to two other health areas in the same health district that did not receive SMC that same year. The health areas in question cover a total of 33 villages, 17 in the SMC implementation area and 16 in the control area with an estimated population of 25,075 and 37,850 inhabitants in 2015, respectively.

The health district of Ouelessebougou has a reference Health Centre (district hospital) and a community Health Centre (CSCOM) in each of the 16 health sub-districts. In each sub-district, there is a health technician or physician called the centres technical director, who works closely with community health workers (CHWs) who are located in villages more than 5 km from the CSCOM. These CHWs were trained in the management of uncomplicated malaria diagnosed by rapid diagnostic tests (RDTs) at the community level. Severe malaria cases were referred to the CSCOM.

### Study design

This is a historical cohort type study. Children in the two health sub-districts where SMC was implemented during the malaria transmission season in 2015 were considered in this study as the intervention group. The intervention consisted of administration of SP–AQ in children aged 3 to 59 months at monthly intervals during the high malaria transmission season from August to November 2015. The control group consisted of children from the other two health sub-districts where SMC was not implemented during that same period.

### Sampling

The sampling covered all children in the four health areas (two who had received SMC in 2015 and two who had not received SMC in 2015). A questionnaire-based household interview survey was used to collect data on hospitalizations and death of children under 5 years of age. The information collected included locality, health area, household number, number living in the household, date of birth, hospitalizations (including dates, causes and number of hospitalizations), and vital status (death or non-death, including the date) and completed with the information on hospitalizations and deaths from the health centres records.

### Sample size

The sample size was calculated on the basis of Mali’s previous mortality rate data documented as 95 per 1000 in children under 5 in 2014 [5]. Using this rate, to detect at least 25% reduction in morality in the group of children under 5 who receiving SMC with 80% power at 5% significance level and missing information of up to 10%, about 2681 children under 5 years of age per group were needed.

### Ethical and regulatory aspects

An amendment to include this evaluation in a study protocol already approved by the Ethics Committee of the Faculty of Medicine of Pharmacy and Dentistry of the University of Technical Sciences, Techniques and Technologies of Bamako (USTTB) was done. The amendment was submitted same Ethic Committee and approved before the start of investigations. Community permission was obtained from village chiefs and community representatives, and informed consent from each household before data collection.

### Data collection

The survey was conducted in February 2017. A data collection questionnaire was developed on tablets with Open Data Kit (ODK) software. The data was entered in ODK and sent to a server before being exported for correction and analysis. Three days of training was provided beforehand to the enumerators on the data collection tools. For hospitalization data, a separate questionnaire was developed. The information collected for each hospitalization included the date of hospitalization, the place of hospitalization, the cause of hospitalization, and the outcome of hospitalization for each child. Information on the date of death, the place of death and the cause of death was collected for each deceased child in order to calculate the follow-up time for each child.

### Data analysis

Pearson’s Chi squared test was used for the comparison of proportions. All tests were bilateral and a value of p < 0.05 was considered significant. The incidence rates of hospitalizations (the number of hospitalizations divided by the total average population of children under 5) and mortality in both groups were expressed per 1000 children per year with confidence intervals of 95%. Dependent variables were represented by deaths and hospitalizations expressed in categorical variables. A negative binomial regression model was used to compare hospitalization rates in both groups after adjusting for age and gender. Data processing and statistical analysis were performed on R version 3.6.1 (https://www.r-project.org; R Foundation for Statistical Computing; Vienna, Austria) and Stata version 14.0 (https://www.stata.com/stata14/; StataCorp LLC, College Station, Texas, USA).

## Results

### Baseline characteristics

This study covered 6638 children under 5 years of age including 2759 children in the SMC intervention group and 3879 children in the control group. Median age was 34.4 months in the intervention group and 35.4 in the control group. The boys represented 52.1% in the intervention group and 50.9% in the control. There was no statistically significant difference between the two groups in term of age and gender distribution (Table 1).

**Table 1:** Distribution of study children by age, sex, and group

	SMC	No SMC	*p* value
	(n = 2759)	(n = 3879)	
Gender, % male	52.1	50.9	*0.327*
Median age in months (interquartile ranges)	36.4 (23.9–47.8)	35.4 (24.2–46.7)	*0.277*

No SMC, control group; SMC, intervention group

### Incidence of all-cause hospitalizations, severe malaria and mortality

The incidence rate of all-cause hospitalizations, severe malaria and death are presented in Table 2. The incidence rate of all causes hospitalizations was 19.60 per 1000 person-years in health areas that received SMC versus 33.45 per 1000 in health areas that did not receive SMC with an incidence rate ratio (IRR) of 0.58 (95% CI 0.41–0.81, p < 0.001). After adjusting for age and gender using a negative binomial regression model, the incidence rate ratios (IRR) remained unchanged 0.61 (95% CI 0.44–0.84, p = 0.003), corresponding to a 39% reduction in hospitalizations for all causes.

**Table 2:** Incidence of all-cause hospital admissions, hospital admissions for severe malaria and all-cause mortality by group

	SMC−		SMC+		IRR		IRR	
	Numbers of Episodes (child-years)	Incidence rate per 1000 person-years	Numbers of Episodes (child-years)	Incidence rate per 1000 person-years	Unadjusted (95% CI)	p-value	Adjusted (95% CI)^a^	p-value
All-cause Hospital admissions	129 (3856.222)	33.45	54 (2754.077)	19.60	0.58 (0.41–0.81)	*< 0.001*	0.61 (0.44–0.84)	*0.003*
Hospital admissions for severe malaria	98 (3856.222)	25.41	36 (2754.077)	13.07	0.51 (0.34–0.76)	*< 0.001*	0.53 (0.36–0.79)	*0.002*
All-cause Mortality	32 (3856.222)	8.29	10 (2754.077)	3.63	0.43 (0.19–0.91)	*0.022*	0.44 (0.22–0.91)	*0.002*

SMC*−*, control group; SMC+, intervention Group

^a^Adjusted for age and gender

The incidence rates of hospitalizations due to severe malaria, were 13.07 per 1000 person-years in the intervention area compared to 25.41 per 1000 person-years in the control area, with an unadjusted relative risk of 0.51 (95% CI 0.34–0.76, p < 0.001). After adjusting for age and gender, the incidence rate ratio remained very similar IRR = 0.53 (95% CI 0.36–0.79, p = 0.002) corresponding to a 47% reduction in hospitalizations related to severe malaria.

The all-cause mortality rate was 3.63 per 1000 person-years in the intervention group and 8.29 per 1000 population in the control group corresponding age and gender adjusted hazard ratio of 0.44 (95% CI 0.22–0.91, p = 0.002). This corresponds to a reduction in mortality of 66% in the intervention group.

## Discussion

This study found that SMC implementation was associated with a substantial reduction in on all-cause mortality and in all cause hospital admissions and hospital admissions due to severe malaria. The findings on all-cause hospitalization show a substantial impact of SMC implementation. This 39% reductions in all cause hospital admissions is consistent with the 41% of all-cause hospital admissions by Wilson et al. in a meta analysis of the efficacy trials [3]. In the district of Kita, Mali a higher reduction 72% in number of severe malaria reported in the health centres in under 5 years of age, during the 2014 transmission season when SMC was implemented compared the 2013 season when SMC was not implemented [7].

For severe malaria hospitalizations, the study found a relative risk IRR of 0.53 (95% CI 0.36–0.79) corresponding to a 47% reduction of malaria-related hospitalizations in the intervention area. This result is lower than that found in Wilson’s meta-analysis and the Cochrane review, which reported a 73% reduction in the incidence of severe malaria cases in a clinical trial [3, 8, 10]. This difference may be explained by the fact that our study was carried out in the context of suboptimal implementation of SMC with coverage. Results based on the predictive estimates for the impact of SMC on episodes of hospitalization and malaria mortality in children found that high coverage of treatment provides proportionally higher protective efficacy for severe episodes malaria and mortality due to malaria [4]. However, it is possible that some environmental factors (parasite circulation and number of larval sites) were not taken into account in the analysis and may have had an effect on our results.

There was a 66% reduction in all-cause mortality areas that received SMC versus those that did not receive SMC in this study. This is consistent with the 57% reduction in all-cause mortality that was found in a meta-analysis of clinical trials [3]. The specific death rate for all causes in this study was 3.63 per 1000 children years, which is higher than the results reported in the Cochrane review of 2 per 1000 children years [9]. In Senegal, although SMC had been given to children over a 3-year period, a study by Cisse et al. did not find a difference in mortality between SMC and non-SMC areas [10]. The authors reported that this may be due to the reduction in malaria transmission associated with scaling up of universal coverage of LLINs and the increased use of antibiotics in children with negative malaria rapid diagnostic test. The combination of the insecticide-impregnated nets and chemoprevention are known to provide additive protective effect against malaria [11].

The high effectiveness of SMC in protecting against hospital admission and deaths is consistent with the low prevalence molecular markers associated with resistance to SP and AQ in study area in Ouelessebougou [12] and the high in vivo efficacy of SP+AQ in the neighboring district of the Bougouni in Mali [13].

Limitations of this study include the retrospective assessment of outcomes that could be subject to recall bias. Although the heath sub-districts were SMC was implemented in 2015 were selected randomly and control areas selected were those contiguous to intervention areas and most similar, a difference in incidence of the outcomes due to other factors than SMC such as baseline malaria risk and treatment-seeking and prevention behaviours could not be excluded. Additional evaluations ongoing on ACCESS–SMC countries and in Senegal would provide more insights on the effectiveness of this important strategy on these outcomes.

## Conclusion

SMC implementation was associated with a substantial reduction in hospital admissions and all-cause mortality. The supports a wide implementation of the strategy to reduce malaria burden in Sahelian countries.

## Data Availability

The corresponding author had full access to all the data in the study and data are available at request to the corresponding author.

## Abbreviations

AQ: Amodiaquine
IRR: Incidence rate ratio
MRTC: Malaria Research and Training Center
ODK: Open Data Kit
SMC: Seasonal malaria chemoprevention
SP: Sulfadoxine–pyrimethamine
USA: United States of America
USAID: United States of America Agency for International Development
WHO: World Health Organization
